# The Hippo Signaling Pathway in Cancer: A Cell Cycle Perspective

**DOI:** 10.3390/cancers13246214

**Published:** 2021-12-10

**Authors:** Yi Xiao, Jixin Dong

**Affiliations:** Eppley Institute for Research in Cancer and Allied Diseases, Fred & Pamela Buffett Cancer Center, University of Nebraska Medical Center, Omaha, NE 68198, USA; yi.xiao@unmc.edu

**Keywords:** cell cycle, checkpoint, cancer, Hippo pathway, phosphorylation

## Abstract

**Simple Summary:**

Cancer is increasingly viewed as a cell cycle disease in that the dysregulation of the cell cycle machinery is a common feature in cancer. The Hippo signaling pathway consists of a core kinase cascade as well as extended regulators, which together control organ size and tissue homeostasis. The aberrant expression of cell cycle regulators and/or Hippo pathway components contributes to cancer development, and for this reason, we specifically focus on delineating the roles of the Hippo pathway in the cell cycle. Improving our understanding of the Hippo pathway from a cell cycle perspective could be used as a powerful weapon in the cancer battlefield.

**Abstract:**

Cell cycle progression is an elaborate process that requires stringent control for normal cellular function. Defects in cell cycle control, however, contribute to genomic instability and have become a characteristic phenomenon in cancers. Over the years, advancement in the understanding of disrupted cell cycle regulation in tumors has led to the development of powerful anti-cancer drugs. Therefore, an in-depth exploration of cell cycle dysregulation in cancers could provide therapeutic avenues for cancer treatment. The Hippo pathway is an evolutionarily conserved regulator network that controls organ size, and its dysregulation is implicated in various types of cancers. Although the role of the Hippo pathway in oncogenesis has been widely investigated, its role in cell cycle regulation has not been comprehensively scrutinized. Here, we specifically focus on delineating the involvement of the Hippo pathway in cell cycle regulation. To that end, we first compare the structural as well as functional conservation of the core Hippo pathway in yeasts, flies, and mammals. Then, we detail the multi-faceted aspects in which the core components of the mammalian Hippo pathway and their regulators affect the cell cycle, particularly with regard to the regulation of E2F activity, the G1 tetraploidy checkpoint, DNA synthesis, DNA damage checkpoint, centrosome dynamics, and mitosis. Finally, we briefly discuss how a collective understanding of cell cycle regulation and the Hippo pathway could be weaponized in combating cancer.

## 1. Introduction

### 1.1. The Cell Cycle and Its Dysregulation in Cancer

The cell cycle is a highly ordered and precise process that ensures the equal distribution of genetic materials to two daughter cells. The cell cycle is divided into four phases: G1 phase (gap1 phase), S phase (synthesis phase), G2 phase (gap2 phase), and M phase (mitosis) [1,2]. When cells exit the cell cycle, they will enter the quiescent state known as G0. In G1, mitogenic signals stimulate the expression of D-type cyclins (D1, D2, and D3), which bind and activate their catalytic partners CDK4 and CDK6 [3,4]. The activated holoenzyme Cyclin D-CDK4/6 phosphorylates the retinoblastoma protein (pRb), leading to the partial activation of the E2 factor (E2F), a key transcription factor that pushes the cells through the restriction point (R point) by up-regulating genes critical for cell cycle transition (e.g., Cyclin D1, Cyclin D3, Cyclin E1/2, CDK2, Cyclin A1/2, PLK1) and DNA synthesis (e.g., Cdc6, Cdc45L, MCM2-7) [5,6,7,8]. Phosphorylation of pRb by Cyclin D-CDK4/6 then primes the further phosphorylation of E2F by Cyclin E-CDK2, which unleashes full E2F transcriptional activity [9]. Cyclin E-CDK2 also propels the G1/S transition by stabilizing the replication origin licensing factor Cdc6, facilitating the assembly of the CMG complex (Cdc45, MCM2-7, and GINS), and recruiting other crucial molecules (e.g., MCM10, RECQ4, AND-1) for DNA replication origin firing [10,11]. Upon entering the S phase, Cyclin A-CDK2 replaces Cyclin E-CDK2 to control DNA replication [11,12]. In G2, Cyclin A forms a complex with CDK1, and together with PLK1 and Cdc25, facilitates nuclear localization and boosts the kinase activity of Cyclin B-CDK1 to prepare for mitotic entry [13,14]. Cyclin B-CDK1 is the master of mitosis that regulates several mitotic events such as centrosome separation, rearrangement of spindles, chromosome condensation, etc. [2,15]. Other mitotic kinases, such as PLK1 and Aurora kinases, are also implicated in the mitotic process [15]. There are two groups of CKIs that counteract the activity of CDKs—the INK4 family (p16INK4a, p15INK4b, p18INK4c, and p19INK4d) and the Cip/Kip families (p21CIP1, p27KIP1, and p57KIP2). The INK4 family specifically inhibits Cyclin D-CDK4/6, while the Cip/Kip families are the pan-CDK inhibitors [16]. Another mechanism for monitoring the order and accuracy of the cell cycle is via cell cycle checkpoints, including the G1 tetraploidy checkpoint mediated by p53 and pRb; the DNA damage checkpoint mediated by ATM, ATR, CHK1, CHK2, BRCA1, BRCA2, PARP1, p53, etc.; and the spindle assembly checkpoint mediated by MPS1, MAD1, MAD2, BUB1, BUBR1, etc. [17,18,19,20,21].

Cancer cells are often characterized by ultra-powerful cell cycle “engines” (mainly Cyclins and CDKs) and defective “brakes” (mainly CKIs and many checkpoint molecules) that drive uncontrolled proliferation and genomic instability [22,23]. For example, CDK4, CDK6, CDK2, and their dependent Cyclins, along with PLK1, Cdc25, and Aurora kinases are amplified or overexpressed in various human cancers [22]. In contrast, p16INK4a, p15INK4b, p27KIP1, p57KIP2, and pRb are deleted, mutated, epigenetically repressed, or degraded [22]. The checkpoint proteins such as p53, ATM, CHK2, ATR, BRCA1, BRCA2, BUB1, and BUBR1 are inactivated, whereas PARP1 is overexpressed in tumors [24,25,26]. Given the aberrant status of the cell cycle machinery in human cancers, cell cycle inhibitors have been widely developed, tested, and utilized in the clinic [19,27]. CDK4/6 inhibitors cause G1 arrest and are used in hormone receptor-positive and HER2-negative metastatic breast cancer [28]. PARP inhibitors can trigger synthetic lethality in the “BRCAness” cases and are used in several types of cancers [29]. Therefore, cell cycle dysregulation is a double-edged sword, which on one hand leads to an aggressive cancer phenotype, yet on the other hand provides targets for anti-cancer therapies.

### 1.2. The Hippo Pathway and Its Dysregulation in Cancer

**Figure 1:**
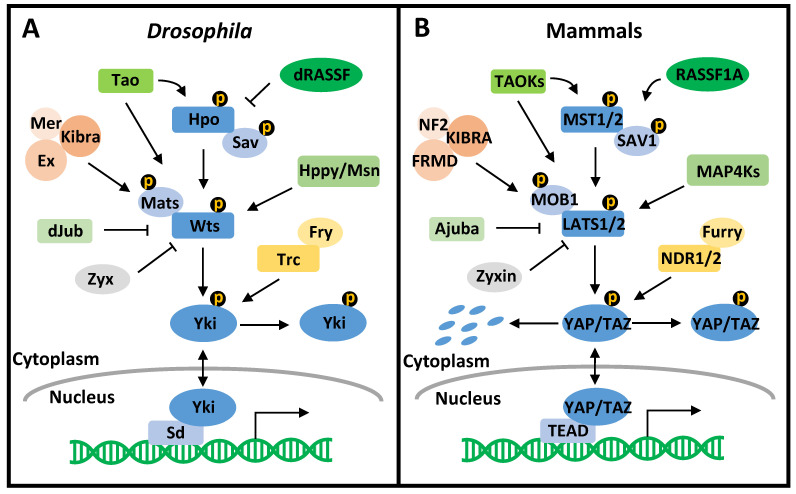
The Hippo pathway in *Drosophila* and Mammals. (**A**) The *Drosophila* Hippo pathway. The core kinase cascade of the *Drosophila* Hippo pathway consists of Hpo, Sav, Wts, Mats, and Yki. When the Hippo pathway is activated, Yki is phosphorylated and sequestered in the cytoplasm. Otherwise, Yki localizes to the nucleus and binds Sd to stimulate gene transcription. Many elements are involved in the direct interaction with the core components to regulate the *Drosophila* Hippo pathway, including Tao, dRASSF, Kibra–Ex–Mer complex, Hppy/Msn, dJub, Zyx, and Fry–Trc complex. (**B**) The mammalian Hippo pathway. The core of the mammalian Hippo pathway is comprised of MST1/2, SAV1, LATS1/2, MOB1, and YAP/TAZ. When the mammalian Hippo pathway is on, YAP/TAZ are phosphorylated and are either retained in the cytosol or targeted for degradation. When the mammalian Hippo pathway is off, YAP/TAZ translocate to the nucleus to activate TEAD for gene transcription. The extended elements in the mammalian Hippo pathway include TAOKs, RASSF1A, KIBRA–FRMD–NF2 complex, MAP4Ks, Ajuba, Zyxin, and Furry-NDR1/2 complex.

The Hippo pathway is a highly conserved signaling pathway that controls organ size, tissue homeostasis, and cancer development (Figure 1) [30]. The Hippo pathway was first characterized in *Drosophila melanogaster,* with the core components consisting of the Ste20-like protein kinase Hippo (Hpo), the WW domain-containing scaffold protein Salvador (Sav), the nuclear dbf2-related (NDR) family protein kinase Warts (Wts), the adaptor protein Mob as tumor suppressor (Mats), and the transcriptional coactivator Yorkie (Yki) [31,32,33,34,35]. Their corresponding mammalian orthologs are mammalian sterile 20-like 1/2 (MST1/2), Salvador homolog 1 (SAV1), large tumor suppressor kinase 1/2 (LATS1/2), MOB kinase activator 1A/B (MOB1A/B), and Yes-associated protein (YAP)/transcriptional co-activator with PDZ binding motif (TAZ) [36,37,38,39,40,41]. The Hippo pathway is activated by a series of phosphorylation events [42,43,44]. In short, stimulated by upstream signals, MST1/2 (Hpo) is phosphorylated and further phosphorylates SAV1 (Sav) and MOB1A/B (Mats) to assist in the recruitment of LATS1/2 (Wts). Phosphorylated by MST1/2 (Hpo), LATS1/2 (Wts) directly phosphorylates YAP/TAZ (Yki), leading to the cytoplasmic retention or degradation of YAP/TAZ (Yki). Inactivated YAP/TAZ (Yki) fails to bind the transcription factor TEA domain-containing protein (TEAD (Sd)), resulting in the repressed transcriptional state of TEAD (Sd) by association with Vestigial-like 4 (VGLL4 (Tgi)). Conversely, the inactivation or degradation of MST1/2 (Hpo), SAV1 (Sav), LATS1/2 (Wts), and MOB1A/B (Mats) results in the nuclear localization of YAP/TAZ (Yki) that competes with VGLL4 (Tgi) to bind TEAD (Sd) and increase the expression of pro-cancerous genes. Over the years, the Hippo pathway has been expanded to incorporate more elements that affect the activity of MST1/2 (Hpo), LATS1/2 (Wts), or YAP/TAZ (Yki). For example, TAO protein kinases (TAOKs (Tao)) activate the Hippo pathway by directly phosphorylating MST2 and LATS1/2 [45,46]. While mammalian RASSF1A promotes MST2 autophosphorylation and increases the kinase activity of MST1 [47,48], *Drosophila* RASSF antagonizes Hpo by interfering with Hpo–Sav interaction [49]. The MAP4Ks (Hppy/Msn) act in parallel to MST1/2 to phosphorylate and activate LATS1/2 [50]. The KIBRA–FRMD–NF2 complex (Kibra–Ex–Mer complex) recruits LATS1/2 to the plasma membrane to facilitate LATS1/2 phosphorylation by MST1/2-SAV1 [51,52,53,54]. Ajuba (dJub) associates with LATS1/2-SAV1 to dampen YAP phosphorylation, and Zyxin (Zyx) activates YAP by facilitating LATS2 degradation [55,56,57]. NDR1/2 (Trc), in complex with Furry (Fry), phosphorylates YAP and promotes its cytoplasmic retention [58,59]. Although there is still a plethora of other participants in the Hippo pathway [60], they are not discussed here due to the scope of this review.

The overexpression of YAP/TAZ is a common feature in a variety of cancers, including glioma, non-small-cell lung cancer, pancreatic cancer, colorectal cancer, sarcoma, melanoma, breast cancer, prostate cancer, etc. [61]. Genetic alteration and epigenetic modification of the Hippo pathway components are also observed in human cancers, such as the YAP amplification, mutation, and deletion of LATS2 and neurofibromatosis type 2 (NF2), and the promoter hypermethylation of LATS2 [62]. Dysregulation of the Hippo pathway leads to hyperactivity of YAP/TAZ, ultimately promoting the transcription of genes involved in cancer survival, proliferation, migration, invasion, immunosuppression, drug resistance, metabolism reprogramming, etc. [60]. In view of the prevalence of the dysregulated Hippo pathway in cancer, compounds have been developed to decrease YAP expression (e.g., CA3), block the interaction between YAP/TAZ and TEAD (e.g., verteporfin, bis-aryl hydrazine scaffold, and VGLL4 peptide), or target the palmitoylation pocket of TEAD (e.g., flufenamic acid) [63,64]. The effectiveness of these inhibitors in restraining tumor growth has been validated in cell and/or animal models, and some drugs will be further evaluated in clinical trials. For example, ION537, an antisense oligonucleotide that is designed to reduce the expression of YAP1, will be tested in a phase I clinical trial for molecularly selected advanced solid tumors (NCT04659096). The TEAD inhibitor VT3989 will be examined in a phase I clinical study in patients with advanced pleural malignant mesothelioma and refractory metastatic solid tumors (NCT04665206). Additionally, patients with advanced mesothelioma, and other solid tumors harboring loss-of-function NF2/LATS1/LATS2 genetic alterations or functional YAP/TAZ fusions, are being recruited as candidates for IAG933 in a phase I study, although its mechanism of action has not yet been disclosed (NCT04857372). Given the tight association between Hippo pathway dysregulation and cancer, it will be very exciting for us to witness the day when these drugs finally enter the clinic.

## 2. The Hippo Pathway in Mitosis: From Yeast and Fly to Mammal

### 2.1. The Yeast Hippo Pathway in the Regulation of Mitotic Exit and Cytokinesis

#### 2.1.1. MEN and RAM: The Hippo Pathway of Saccharomyces Cerevisiae 

The Hippo pathway is conserved among the eukaryotic kingdom to regulate mitotic exit (Table 1). This part of this review will mainly describe the similarity of the yeast Hippo pathway to the mammalian Hippo pathway since the conservation between *Drosophila* and mammals has been addressed in the previous section. The budding yeast *Saccharomyces cerevisiae* uses the Cdc fourteen early anaphase release (FEAR) network, mitotic exit network (MEN), and Ace2 and morphogenesis (RAM) network to assist M/G1 transition [65], among which MEN and RAM are conserved in the *Drosophila* and mammalian Hippo pathways. FEAR and MEN coordinate the metaphase-to-anaphase transition, and RAM facilitates cell separation [65].

The core of the MEN includes the GTPase Tem1, protein kinase Cdc15 (MST1/2 in mammals), scaffold protein Nud1 (SAV1 in mammals), protein kinases Dbf2/20 (LATS1/2 in mammals), adaptor protein Mob1 (MOB1A/B in mammals), and the effector phosphatase Cdc14 [65]. In the anaphase, GTP-binding Tem1 recruits Cdc15 to the spindle pole bodies (SPBs, centrosomes in mammals), where the MEN machinery is assembled [65]. Cdc15 phosphorylates Nud1 to provide an acidic docking site for the binding of the Dbf2–Mob1 complex through the basic pocket of Mob1, which brings Dbf2–Mob1 in close proximity to Cdc15 for phosphorylation [66,67,68]. The phospho–Dbf2–Mob1 complex then phosphorylates Cdc14 to block its nuclear localization signal, resulting in the re-localization of Cdc14 from the nucleus to the cytosol. In the cytosol, Cdc14 antagonizes mitotic Clb-Cdc28 (Cyclin B-CDK1 in mammals) activity, including activating the Cdc28 inhibitor, promoting Clb degradation, and dephosphorylating substrates of Clb-Cdc28, to drive the mitotic exit process [69,70,71,72]. Although highly conserved, the MEN in budding yeast differs from the mammalian Hippo pathway in that mammalian MST1/2 is activated via phosphorylation, but in *Saccharomyces cerevisiae*, non-phosphorylatable mutant Cdc15 more effectively inactivates the mitotic Clb–Cdc28 complex [73]. Another disparity is that in mammals, the downstream effectors YAP/TAZ are sequestered and degraded in cytosol by LATS1/2-mediated phosphorylation, while in *Saccharomyces cerevisiae*, Cdc14 translocates and functions in cytosol upon Dbf2-mediated phosphorylation.

The core elements of the RAM are the scaffold protein Hym1, the leucine-rich protein Sog2, protein kinase Kic1 (MST1/2 in mammals), scaffold protein Tao3 (Furry in mammals), protein kinase Cbk1 (NDR1/2 in mammals), adaptor protein Mob2 (MOB1A/B in mammals), and the transcription factor Ace2 [65]. The helical armadillo-repeat protein Hym1 allosterically activates Kic1 [74,75]. Sog2 is not conserved among eukaryotes, but it is crucial for the localization of the Kic–Hym1 complex to the bud neck during late mitosis where Kic1 directly phosphorylates Cbk1 at the hydrophobic motif site [75,76]. Interaction between Kic1 and Cbk1 is further promoted by Tao3 and Mob2 [77]. As binding with Mob1 is required for Dbf2 kinase activity in MEN, Mob2 is necessary for Cbk1 kinase activity in RAM [68,78]. Activated Cbk1 then phosphorylates Ace2 to facilitate Ace2 nuclear localization in the daughter cell, leading to the increased transcription of Ace2-targeted genes (e.g., CTS1, SCW11, WHR143W, and YER124C) to control cell separation [79,80,81]. Although the phosphorylation modification has been extensively studied in the mammalian Hippo pathway, it is still unclear whether Kic1, Tao3, and Mob2 are also modified through comparable mechanisms. Future work may shed some light on the phosphorylation regulation of these components to provide a more comprehensive understanding of the RAM network.

#### 2.1.2. SIN: The Hippo Pathway of Schizosaccharomyces Pombe

In the fission yeast *Schizosaccharomyces pombe*, the Hippo pathway is conserved in the septation initiation network (SIN, in parallel with *Saccharomyces cerevisiae* MEN), which *Schizosaccharomyces pombe* uses to initiate septum formation. The morphogenesis Orb6 (MOR, in parallel with the *Saccharomyces cerevisiae* RAM) network is essential for the polarized growth of *Schizosaccharomyces pombe* at the interphase [82,83]. As MOR is not mainly involved in mitosis regulation, we will not further discuss it in the following sections.

The SIN machinery comprises the GTPase Spg1, protein kinase Cdc7 (MST1/2 in mammals), scaffold protein complex Cdc11–Sid4–Ppc89 (Cdc11 is similar to SAV1 in mammals), protein kinase Sid1, adaptor protein Cdc14 (unrelated to Cdc14 of *Saccharomyces cerevisiae*), protein kinase Sid2 (LATS1/2 in mammals), adaptor protein Mob1 (MOB1A/B in mammals), and the effector phosphatase Clp1, also known as Flp1 [84]. When the spindle is well-assembled at the metaphase, the bipartite GTPase-activating protein (GAP) Cdc16-Byr4 dissociates from SPB, allowing for Spg1 activation and Cdc7 recruitment to both SPBs [85,86]. At the onset of the anaphase, Cdc16-Byr4 is again recruited to one of the SPBs to displace Cdc7, giving rise to an asymmetric distribution of Cdc7 at SPBs [86,87]. Tethered directly to the SPB(s) is the scaffold protein complex consisting of Cdc11–Sid4–Ppc89 [84]. Sid4 is anchored to the SPB via binding with the constitutively localized protein Ppc89 at the C-terminus, and the N-terminus of Sid4 is associated with the scaffold protein Cdc11 that bridges other SIN components to the SPB [88,89,90]. During the anaphase, when Cdc13-Cdc2 (Cyclin B-CDK1 in mammals) activity is low, Sid1 (in complex with Cdc14) localizes to the Cdc7-containing SPB [91]. The proper loading of the upper SIN components sets the stage for the localization and activation of the Sid2–Mob1 complex at the cell division site to promote medial ring constriction and septation [92,93]. With kinase activity peaking at the end of the anaphase, the Sid2-mediated phosphorylation of Clp1 is boosted, which creates the binding sites for the 14-3-3 protein Rad24 to increase the cytoplasmic retention of Clp1, where Clp1 antagonizes Cdc2 activity [92,94,95,96]. Reduced Cdc2 activity further activates SIN signaling that confers robust cytokinesis [95,97,98]. Despite having a kinase cascade closely related to that of the mammalian Hippo pathway, SIN has a distinctive tripartite scaffold complex and an extra set of kinase complex (Sid1–Cdc14). Besides, unlike the mammalian Hippo pathway effector YAP that becomes inactive upon cytoplasmic retention, the *Schizosaccharomyces pombe* Clp1 more closely resembles its *Saccharomyces cerevisiae* ortholog Cdc14 that exerts its functions in the cytosol. There is evidence that Cdc7 is phosphorylated by CDK1 in mitosis and dephosphorylated by PP1α as cells exit mitosis [99]. Thus, similar to its *Saccharomyces cerevisiae* peer Cdc15, Cdc7 is more likely to facilitate mitotic exit in a phosphorylation-free state, which is another difference from its mammalian peer MST1/2 that are activated by phosphorylation. Surprisingly, the scaffold protein of SIN, Cdc11, is phosphorylated and activated by its downstream kinase Sid2, while the mammalian scaffold Sav1 is phosphorylated and activated by the upstream kinase MST1/2 [100,101]. Considering the existing similarity and difference of the so far explored phosphorylation patterns between the *Schizosaccharomyces pombe* and mammalian Hippo pathways, disclosing the phosphorylation regulation in SIN will no doubt be an intriguing subject.

### 2.2. The Conservation of the Drosophila and Mammalian Hippo Pathway in Mitosis

#### 2.2.1. The Drosophila Hippo Pathway in Mitosis

Based on the structural conservation of the Hippo pathway from yeast to higher eukaryotes, it is reasonable to speculate that the functional conservation in mitosis also exists in the *Drosophila* Hippo pathway as well as the mammalian Hippo pathway. Indeed, the *Drosophila* Hippo components Hpo, Sav, Wts, and Mats are involved in mitosis. Hpo deficiency leads to chromosome misalignment and spindle defects, which in turn activates spindle assembly checkpoint (SAC) to delay mitotic exit [102,103]. Wts controls spindle orientation by phosphorylating the spindle pole protein Mud to enhance the affinity of Mud to the spindle positioning regulator Pins, and the knockdown of Hpo, Sav, or Wts causes the misorientation of the spindle [104]. Moreover, Wts depletion contributes to a higher incidence of polyploid cells, a sign of cytokinesis failure [105,106]. The Wts co-activator Mats localizes at the centrosome as well during mitosis and is required for proper chromosome segregation [107]. In addition, a gain-of-function screen has identified Mats as a cytokinesis regulator [108]. The overexpression of Mats suppresses the cytokinesis failure-related rough eye phenotype induced by dominant negative Pbl [108]. Interestingly, Hpo-Wts signaling reduces the level of Rae1, a microtubule-associated protein necessary for spindle assembly [109,110]. Therefore, too much Hpo-Wts signaling might phenocopy the loss of Hpo or Wts, resulting in the disruption of spindle homeostasis and an aberrant mitotic outcome. Notably, the NDR kinase signaling Fry-Trc more functionally resembles MOR of *Schizosaccharomyces pombe*, not SIN of *Saccharomyces cerevisiae*, to regulate polarized cell growth rather than mitosis [111]. Although there is no direct evidence demonstrating that Yki can affect mitosis, Yki localizes to the mitotic chromatin [112]. Given that Yki recruits P-TEFb to promote the transcriptional pause release and inhibition of P-TEFb in mitosispostpones cell cycle progression [113,114], it is plausible that Yki mediates M/G1 transition by facilitating transcriptional pause release during mitosis. 

#### 2.2.2. The Mammalian Hippo Pathway in Mitosis

All the mammalian counterparts of the yeast Hippo pathway have been shown to regulate mitosis. Both the expression and the kinase activity of MST1/2 are elevated in mitosis [39]. The MST1-NDR1 pathway inhibits the hyperactivation of Aurora B, and the suppression of MST1 or NDR1 afflicts kinetochore-microtubule attachment [115]. MST2 is phosphorylated by CDK1 at S385 in mitosis, and the MST2-Furry-NDR1 pathway is crucial for chromosome alignment [116,117]. Furthermore, the MST1/2-SAV1-Nek2A pathway cooperates with motor protein kinesin-5 to regulate bipolar spindle formation [118].

The kinase activity of LATS1 is specifically increased in mitosis [119]. LATS2 translocates to the nucleus with elevated expression upon nocodazole-induced mitotic arrest [120]. The ectopic expression of LATS1 prevents mitotic entry by downregulating Cyclin A and Cyclin B [121,122]. LATS1 also counteracts PLK1 to delay mitotic entry in response to ionizing radiation-induced genotoxic stress [123]. LATS2 overexpression gives rise to G2/M arrest via increasing the inhibitory phosphorylation of CDK1 at Y15 [124]. LATS1 is phosphorylated by CDK1 at T490 and S613 in mitosis and is enriched in spindle, spindle midzone, and midbody, depending on the different stages of mitosis [125,126,127]. LATS2 is phosphorylated by CDK1 at S157, S342, T349, S598, and S1027 and by Aurora A at S380 in mitosis [128,129]. Besides, LATS1/2 phosphorylates Aurora B in vitro [129]. Similar to LATS1, LATS2 accumulates at the mitotic apparatus during mitosis [130]. The overexpression of LATS1 accelerates the mitotic exit of nocodazole-arrested mitotic cells, and the depletion of LATS1 or MOB1A prolongs the telophase [131]. Furthermore, LATS1 inactivation leads to the persistent activation of SAC followed by mitotic slippage, and the N-terminal truncation of LATS1 induces chromosome misalignment, chromosome mis-segregation, and the ultimate formation of multinucleated cells [119,132]. Similarly, LATS2 deficiency contributes to the loss of mitotic regulators as well as aberrant mitotic events, telophase delay, and cytokinesis failure [133,134,135]. Mechanistically, the LATS1/2-mediated phosphorylation of INCENP at S894 is involved in Aurora B activation, telophase progression, and cytokinesis [133]. One study demonstrates that LATS1/2 phosphorylates CHO1 at S716, which promotes the binding and activation of LIMK1 at centrosome to trigger cytokinesis initiation [136]. However, another study indicates that LATS1 inhibits the kinase activity of LIMK1 at the contractile ring to control cytokinesis [137]. Such a discrepancy could be due to the differential regulation of LATS1/2 on LIMK1, depending on their subcellular localization where LATS1/2-LIMK1 interaction occurs.

MOB1A, along with NDR1 and MOB2, modulates mitotic spindle orientation [138]. MOB1A/B-depleted cells display the delayed localization of mitotic exit orchestrators MKLP2 and CPC to the spindle midzone in the early anaphase [139,140]. Besides, MOB1A silencing leads to a prolonged telophase [131]. Deprivation of MOB1A/B gives rise to abscission failure during cytokinesis [141]. MST1/2-dependent phosphorylation of MOB1A/B also affects mitosis-to-G1 transition [39].

Furry promotes the kinase activity of PLK1 and the depletion of Furry results in chromosome misalignment and multipolar spindle [142]. Additionally, Furry enhances the acetylation of spindle microtubules [143]. As Furry shares a similar localization pattern with LATS1 [116,126], it prompts us to speculate that the MST–SAV1–LATS axis and the MST–Furry–NDR axis may coordinate to regulate mitotic progression. In addition, the activation of the NDR2/GEFH-1/RhoB/YAP axis as a result of RASSF1A silencing induces cytokinesis failure in lung cancer cells [144].

YAP and/or TAZ drives the expression of mitotic genes such as Cyclin A2, Cyclin B2, Aurora B, Cdc25A, CENPF, CdcA5, etc. [145,146,147,148]. Moreover, YAP augments the expression of mitotic genes by synergistically acting with AP-1 [145]. YAP also increases the B-MYC mRNA level and reinforces B-MYC’s binding to the promoter region to induce G2/M gene expression [149]. Furthermore, YAP induces the transcription of E2F as well as FOXM1 to cooperatively stimulate the expression of mitotic genes [150,151,152,153]. Resultantly, YAP elimination triggers G2/M arrest [154]. Both YAP and TAZ are phosphorylated by CDK1 at multiple sites during mitosis [155,156]. Intriguingly, while the mitotic phosphorylation mimicry of YAP contributes to multipolar division, the non-phosphorylatable TAZ mutant is associated with multipolar spindle [155,156]. YAP is also required for SAC activation by the up-regulation of BubR1 [157]. YAP localizes to the spindle zone and midbody ring at the late stage of mitosis and interacts with polarity protein PATJ [158]. YAP deficiency brings about disoriented spindle and delayed abscission, as well as defective cytokinesis [158].

The conservation of human Cdc14 (hCdc14: hCdc14A, hCdc14B, and hCdc14C) [159,160] and yeast Cdc14 (Cdc14 in *Saccharomyces cerevisiae* and Clp1 in *Schizosaccharomyces pombe*) raises two extra questions: 1) Does human Cdc14 regulate mitotic exit? 2) Is Cdc14 downstream of the Hippo pathway in mammals? If not, is there a crosstalk between them? For the former question, the answer seems to be YES at the first glance. Human Cdc14B accumulates to the midzone at the anaphase, the midbody at the telophase or cytokinesis, and the intracellular bridge at the termination of cytokinesis [161], highly resembling the localization pattern of yeast Cdc14 or Clp1 at the later stages of mitosis. The hCdc14A and/or hCdc14B could also rescue the phenotype of the Cdc14 mutant or the Clp1 mutant in yeast [159,162]. The hCdc14A dephosphorylates Cdh1 to enhance activity of the anaphase-promoting complex, APC^Cdh1^ [163]. Additionally, hCdc14A counteracts the CDK1-mediated phosphorylation of kinesin-like protein MKLP1 to allow for central spindle assembly at the anaphase [164,165]. The overexpression of hCdc14A contributes to chromosome mis-segregation and cytokinesis failure [166]. The hCdc14 dephosphorylates Cdc25 to regulate CDK1 activity in mitosis, and the depletion of hCdc14 leads to multipolar spindle, lagging chromosomes, and improper cytokinesis [167]. Nevertheless, the role of hCdc14 in mitotic exit is still controversial. There are studies indicating that abrogation of either hCdc14B or hCdc14A, or the double knockout of hCdc14A and hCdc14B, fails to trigger spindle defects, delayed mitotic exit, or cytokinesis failure [168,169]. Human cells harbor more heterogeneity than yeast. The mitotic regulation is more complicated as well. It is possible that hCdc14 is crucial in the cells that rely heavily on hCdc14 to progress through mitosis, but it is dispensable in the cells that possess more compensatory mechanisms in mitotic exit regulation. The latter question is still open, since there is no evidence suggesting that hCdc14 is regulated by the mammalian Hippo pathway, and studies concerning the interaction between hCdc14 and Hippo pathway components are very limited. The hCdc14A and hCdc14B dephosphorylate the CDK1-mediated mitotic phosphorylation of YAP [155,157]. The hCdc14A and hCdc14B also reverse the CDK1-dependent phosphorylation of kidney and brain enriched (KIBRA) in mitosis, and phosphorylation-deficient KIBRA results in a decreased cell proportion in the G2/M phases [170]. Future research is warranted to sort out a more satisfying answer to the second question.

## 3. The Core of Mammalian Hippo Pathway in Other Phases of the Cell Cycle

### 3.1. MST1/2 in G1 Tetraploidy Checkpoint, DNA Damage Checkpoint, and Centrosome Dynamics

**Figure 2:**
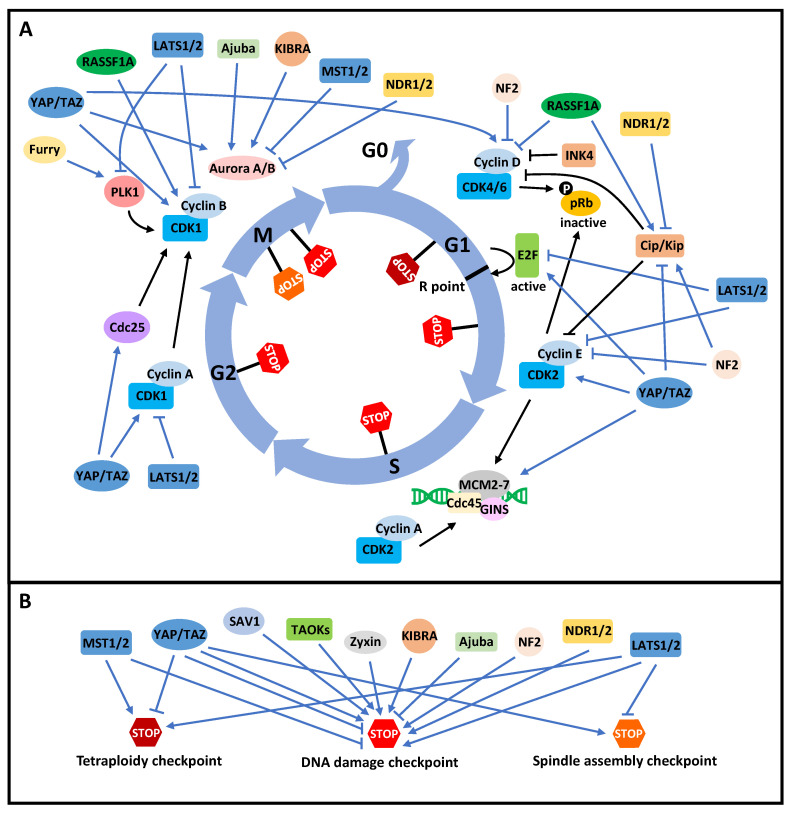
The mammalian Hippo pathway in cell cycle control. (**A**) The regulation of the cell cycle machinery by the Hippo pathway. The intra-cell cycle regulation is indicated by black arrows, and the regulation of Hippo pathway on the cell cycle elements is shown in blue arrows. Without the mitogenic signal, the cells enter the quiescent state, G0. With the stimulation of the mitogenic signal, the cells enter the G1 and continue the cell cycle progression. In G1, Cyclin D-CDK4/6 and Cyclin E-CDK2 phosphorylate pRb to dissociate it from E2F. Activated E2F transcribes cell cycle-related genes and facilitates the passing through of the cells to the R point. The INK4 family inhibits the activity of Cyclin D-CDK4/6. The Cip/Kip family suppresses both Cyclin D-CDK4/6 and Cyclin E-CDK2. Cyclin E-CDK2 and Cyclin A-CDK2 are required to initiate DNA synthesis and control DNA replication during the S phase. In G2, Cyclin A-CDK1, together with Cdc25 and PLK1, activates Cyclin B-CDK1 for mitotic entry. Cyclin B-CDK1, PLK1, and Aurora A/B are the key kinases for mitosis. The Hippo components are highly involved in regulating the activities of CDKs, CDK inhibitors, DNA synthesis factors, and other cell cycle kinases as well as phosphatases to control cell cycle progression, as illustrated by the blue sharp arrows (activation) and blue blunt arrows (inhibition). (**B**) The regulation of the cell cycle checkpoints by the Hippo pathway. As indicated in Figure 2A, there are tetraploidy checkpoints in G1, spindle assembly checkpoints in mitosis, and DNA damage checkpoints throughout the cell cycle. The cell cycle checkpoints monitor the order and accuracy of the cell cycle, and the Hippo pathway components can strengthen (sharp arrow) or weaken (blunt arrow) the checkpoints to regulate the cell cycle process.

The Hippo pathway components have roles in cell cycle control that affect E2F activity, G1 tetraploidy checkpoint, DNA synthesis, DNA damage checkpoint, centrosome dynamics, and mitosis (Figure 2; Table 2). Tetraploidization is the precursor for chromosome instability (CIN), one of the hallmarks of cancer development, and it is monitored through the G1 tetraploidy checkpoint [21,23]. p53 and pRb are the classical gatekeepers that surveil and arrest polyploid cells at G1 [21]. Intriguingly, Hippo signaling is found to be the alternative gatekeeper to prevent the polyploid status [171]. The double knockout of MST1/2 in the liver leads to increased polyploidy, elevated p53 levels, and enlarged liver size as well as hepatic nodule formation, and the triple ablation of MST1/2 and p53 in liver produces an even stronger polyploid phenotype [171].

DNA damage checkpoints arrest cells in response to various extra-cellular and intrinsic DNA damage, allowing time for DNA damage repair to preserve genomic fidelity [172]. There are DNA damage checkpoints in each phase of the cell cycle, and the main players in DNA damage checkpoints are the DNA damage sensors (ATM and ATR), the DNA repairers (BRCA1/2 and PARP1), the mediators (CHK1/2), and the cell cycle arrest or apoptosis executor (p53) [19,172,173]. Genotoxic stress activates the ATR-RASSF1A-MST2-LATS1-CDK2 pathway to reduce the inhibitory phosphorylation of BRCA2 at S3291, thereby promoting the BRCA2-facilitated assembly of the RAD51 nucleofilament at the replication fork to ensure genomic stability [174]. Worthy of note is that MST2 phosphorylates H2B at S14 to shut down rDNA transcription and to promote rDNA repair in response to ionizing radiation-induced DNA injury [175]. In addition, MST2 is required for cell apoptosis that is mediated by the RASSF1A–MST2–LATS1 complex [176]. MST1 also increases p53-induced cell death by inhibiting Sirt1-mediated p53 deacetylation [177].

The centrosome is a microtubule-organizing center (MTOC) and plays versatile roles in cell motility, cell polarity, and the formation of spindle during mitosis [178]. Centrosome cycle, in a simplified description, could be summarized as centriole disengagement in G1, centrosome duplication in S, centrosome maturation in G2, and centrosome separation in mitosis [178,179]. Centrosome dysregulation is associated with genomic instability and cancer [179]. MST1 affects centrosome duplication through the MST1-MOB1-NDR1 pathway [180]. In particular, the kinase activity of MST1, rather than the SARAH domain, promotes centrosome duplication, which means that the interaction between MST1/2 and MST1/2, SAV1, or RASSF is dispensable for this specific function [180,181]. Furthermore, MST1/2, when complexed with SAV1, interacts with Nek2A to facilitate the translocation of Nek2A to the centrosomes where Nek2A phosphorylates and displaces the centrosome linker proteins C-Nap1 and Rootletin from centrosomes, leading to the centrosome disjunction [118].

### 3.2. SAV1 in G1 Tetraploidy Checkpoint, DNA Damage Checkpoint, and Centrosome Dynamics

Liver-specific SAV1 depletion triggers increased polyploid hepatocytes and an enlarged nucleus [171]. Moreover, SAV1, by associating with RASSF1A, activates p73 to induce cell apoptosis, independent of the MST1/2-LATS1/2-YAP pathway [182]. SAV1 is also required in centrosome disjunction, and SAV1 depletion leads to the impaired centrosome translocation of Nek2A and insufficient centrosome separation [118].

### 3.3. LATS1/2 in E2F Activity, G1 Tetraploidy Checkpoint, DNA Synthesis, DNA Damage Checkpoint, and Centrosome Dynamics

E2F activity is modulated by pRb and CDK2/4/6 as well as CDK kinase inhibitors, and E2F activation is required for G1/S transition. The ectopic expression of LATS2 hinders G1/S progression, and LATS2 depletion inhibits pRb-induced senescence [130,183]. LATS2 promotes DREAM (DP, pRB, E2F, and MuvB) complex assembly to repress E2F activity by increasing the DYRK1A phosphorylation of LIN52 [183,184]. Moreover, one study indicates that LATS2 dampens the kinase activity of Cyclin E-CDK2, but the kinase activity of Cyclin D-CDK4/6 seems to be unaltered, nor are the protein levels of CKIs, p21, p27, or p57 significantly changed [185]. Another study, however, shows a decent decrease of Cyclin D and an increase of p27 protein levels in LATS2 downregulated cells [186]. The incongruous observations from these two studies could be due to the disparate cellular background or different experimental settings.

A genome-wide RNAi screen identified LATS2 as a required gene for tetraploid-induced G1 arrest [187]. The enforced expression of LATS2 decreases polyploid formation, whereas the LATS2 depletion or expression of kinase-inactive LATS1 increases polyploid population [119,120,171]. Extra centrosomes in polyploid cells stimulate the activity of Rac1, which antagonizes RhoA, and the reduced activity of RhoA further activates LATS1/2 [187]. LATS1/2 elevates p53 expression, and LATS2 prevents Mdm2-mediated p53 degradation, resulting in the activation of the G1 tetraploidy checkpoint [119,120,121]. The p53 protein, in turn, induces LATS2 at the transcriptional level to suppress tetraploidization [120]. Additionally, LATS2 phosphorylates ASPP1 and promotes its nuclear localization to augment the pro-apoptotic activity of p53 in polyploid cells [188].

There are a few studies linking LATS1/2 to DNA synthesis, but the exact mechanisms concerning how LATS1/2 affects DNA synthesis are still unclear. Although the ectopic expression of LATS2 does not alter the DNA synthesis rate, it retards DNA synthesis initiation [130]. Given that retarded synthesis could be caused by LATS2 overexpression-mediated G1 arrest, it is unknown whether LATS2 indeed modulates the assembly of the pre-replication complex or a sequential phosphorylation process required for the start of DNA replication. Neutral stem cells derived from the brain of LATS1/2 double-knockout mice exhibit a prolonged S phase with increased DNA replication stress as compared with control cells [189]. Cell number-normalized RNA-Seq and gene ontology analysis indicate that “DNA-dependent DNA replication” is enriched in LATS1/2-depleted samples [189], but this study did not explore the detailed mechanism. 

An ATM and ATR substrate analysis reveals LATS1 as one of the substrates in response to DNA damage [190]. The functional analysis further confirms that LATS1 silencing leads to impairment in homologous recombination (HR), G2/M checkpoint activation, intra-S checkpoint activation, and histone H2AX phosphorylation [190]. Consistent with this finding, LATS1 interacts with CDK2 to relieve the inhibitory phosphorylation of BRCA2 by CDK2, which improves DNA repair [174]. Additionally, LATS2 is also induced by ATR-CHK1 signaling in response to mutant H-Ras-generated replication stress, and triggers p53-mediated apoptosis and senescence as well [191].

LATS1 and LATS2 are specifically enriched in the centrosomes during the interphase and most phases of mitosis, and LATS2 is phosphorylated by centrosome kinase Aurora A at S83 [126,134,192,193]. Centrosome overduplication is witnessed in LATS1/2 knockout cells and N-terminal truncated LATS1 mutant-induced cells, while the exogenous expression of LATS2 represses centrosome amplification [132,134,193]. One of the potential mechanisms of LATS1 deficiency-mediated centrosome overduplication is that the loss of LATS1 stabilizes Cdc25B, which confers the hyperactivation of CDK2, and CDK2 phosphorylates centriole duplication licensing factor at T199, resulting in uncontrolled centrosome amplification [193].

### 3.4. MOB1A/B in Centrosome Dynamics

MOB1 accumulates at the centrosome from the interphase to the anaphase of mitosis and localizes to the spindle midzone and midbody at the end of mitosis [141,180]. Compared with other members of the MOB family, the overexpression of MOB1A/B has the most significant effect on centrosome overduplication [180]. The elimination of MOB1A causes reduced centrioles in mitosis, and the induction of shRNA-resistant MOB1A expression in MOB1A-depleted cells rescues the phenotype [180]. Cells deprived of MOB1 are burdened with centrosome disjunction failure as well [141]. In addition, cells with non-phosphorylatable MOB1A are characterized by accelerated progression through G1/S, but the mechanism has not been further explored [39]. However, given the close interaction between MOB1A/B and LATS1/2, it will be interesting to find out whether MOB1A/B, in the same way as LATS1/2, mediates G1/S transition through a similar mechanism, presumably by regulating E2F activity, G1 tetraploidy checkpoint, or DNA damage checkpoint.

### 3.5. YAP/TAZ in E2F Activity, G1 Tetraploidy Checkpoint, DNA Synthesis, and DNA Damage Checkpoint

A couple of studies illustrate that YAP/TAZ abrogation causes G1 arrest [194,195,196,197,198,199]. YAP/TAZ-TEAD directly activates E2F transcription through the binding consensus motif (GGAATG) of the E2F promoter [152,153]. YAP/TAZ indirectly enhances E2F activity by inducing the expression of Cyclin D or Cyclin E, often accompanied by increased protein levels of CDK2/4/6, depending on different experimental conditions [146,148,194,197,198,200,201,202,203,204,205]. YAP/TAZ also negatively regulates p21 or p27 expression to accelerate G1/S transition [205,206,207,208]. A recent study demonstrated that YAP associates with YY1 and EZH2 on the genome to inhibit p27 expression at the transcriptional level [208]. Additionally, in response to mechanical cues, YAP elevates the transcription of ubiquitin ligase Skp2, which subsequently forms a complex with Skp1-Cullin-1-F-box (SCF) to target p21 and p27 for proteasomal degradation [209,210]. By collaborating with FOXM1, an essential cell cycle transcription factor that promotes G1/S progression in part by increasing the Skp2-mediated degradation of p21 and p27, YAP further prompts E2F-mediated gene expression [151,211].

A genetically engineered murine liver model demonstrates that hepatocytes from YAP-induced liver, similar to the LATS1/2-depleted liver, harbors increased sets of genetic materials and an enlarged nucleus [171]. It is proposed that YAP triggers polyploidization through the Akt–p300–Skp2–p27/FOXO axis, in which YAP activates Akt–p300 to promote Skp2 acetylation and cytoplasmic retention, leading to the accumulation of p27 and FOXO degradation [171]. Paradoxically, while YAP diminishes p27 in the cellular context, YAP boosts p27 in the transgenic model. Indeed, p27 or Skp2 ablation in MST1/2-knockout liver decreases the number of multinucleated hepatocytes and diminishes the liver size, yet the phenotype is only partially rescued. Therefore, it is possible that there are targets of YAP that more strongly affect the polyploid phenotype than p27, which await to be explored. In addition, more tissue-specific transgenic models are needed to explain this discrepancy.

YAP-depleted cells start DNA replication more slowly than the control cells after the release from G1 arrest, indicating that YAP is required for replication initiation [212]. YAP elevates the mRNA levels of DNA replication molecules such as Cdc6, MCM2-7, and MCM10 [145,150,151,152,212]. In addition, YAP synergizes with AP-1 and E2F to further augment the transcription of DNA synthesis genes [145,153].

YAP/TAZ promotes DNA repair in response to cisplatin-induced DNA damage by increasing the mRNA levels of corresponding homologous recombination genes such as BRCA1/2, Rad51, and Rad54L [212,213]. How YAP/TAZ regulates p53/p73 to affect the DNA damage response is complicated. On the one hand, TAZ suppresses the expression of p53, and the depletion of TAZ leads to cell cycle arrest and apoptosis [198]. YAP/TAZ restricts the transcriptional activity of p53 by decreasing its acetylation [214,215]. YAP is also capable of binding mutant p53, not wild-type 53, to activated transcription factor NF-Y, and improves the transcription of cell cycle regulators, Cyclin A, Cyclin B, and CDK1 [216]. On the other hand, YAP has been shown to directly increase the transcription of wild-type p53 to enhance chemotherapeutic agent-induced apoptosis in hepatocellular carcinoma [217]. YAP is also able to physically interact with p73 (the homolog of p53) and promote p73-mediated apoptosis by improving p73 transcriptional activity, acetylation modification, and stabilization [218,219,220]. Therefore, given the multiple influences YAP/TAZ could exert on p53/p73, a better way to interpret the outcome is to evaluate the upstream pathway. The phosphorylation of TAZ by LATS1/2 causes TAZ cytoplasmic retention and degradation, which increases p53 acetylation and p53-mediated transcription of pro-apoptotic genes [41,215,221]. However, the LATS1–YAP interaction is diminished when RASSF1A-MST2-LATS1 signaling is stimulated by the ATM-dependent activation of RASSF1A, giving rise to a stronger association between YAP and p73, which promotes apoptosis [176]. Moreover, the c-Abl-mediated phosphorylation of YAP and the PML-mediated sumoylation of YAP also improve YAP stability and augment YAP-p73 signaling in response to DNA damage [222,223]. 

## 4. The Mammalian Hippo Pathway Regulators in Cell Cycle

### 4.1. TAOKs in DNA Damage Checkpoint and Mitosis

TAOKs are phosphorylated and activated by ATM upon genotoxic stress and in turn increase p38 activity to induce G2/M arrest [224]. The kinase activity of TAOKs is triggered during mitosis [225]. TAOKs regulate microtubule stability, and TAOK1 deficiency results in erroneous kinetochore–microtubule attachment [226,227]. TAOK1/2 also affect mitotic cell rounding, spindle positioning, and cytokinesis by phosphorylating Rnd3 (also known as RhoE) [225,228]. Moreover, a high-throughput imaging siRNA assay identified TAOK1 as a critical gene for proper chromosome transmission in mitosis, and TAOK depletion contributes to aberrant mitotic morphology and multiple mitotic defects (including impaired cell rounding, spindle decentralization, chromosome misalignment, chromosome mis-segregation, cytokinesis failure, and prolonged mitosis) [225,227,228]. Interestingly, TAOK1/2 inhibition induces multipolar spindles and mitotic arrest specifically in breast cancer cell lines (SKBR3 and BT549), while nontumorigenic breast cells (MCF-10A) display normal mitosis [229].

### 4.2. RASSF1A in E2F Activity, DNA Damage Checkpoint, Centrosome Dynamics, and Mitosis

Although there is no evidence supporting that RASSF1A directly affects E2F activity, RASSF1A could modulate E2F activity through the regulation of Cyclin D1, CDK4, and p27. The ectopic expression of RASSF1A decreases Cyclin D1 at the protein level and causes G1 arrest, while the absence of RASSF1A leads to Cyclin D1 accumulation [230,231]. RASSF1A also promotes miR-711-mediated CDK4 downregulation and reverses H-RasG12V-induced p27 suppression [232,233].

Activated by ATR in response to DNA lesions, RASSF1A enhances the interaction between LATS1 and CDK2, by which it alleviates the inhibitory effect of CDK2 on BRCA2 to improve DNA repair [174]. DNA damage also triggers the ATM-RASSF1A-MST2 pathway, which promotes MST2 to phosphorylate H2B at S14, and halts rDNA transcription for rDNA repair [175]. In addition, RASSF1A intrinsically associates with the DNA repair protein XPA to augment DNA repair [234]. RASSF1A increases p53 stability by promoting the self-ubiquitination-induced degradation of MDM2 through the disruption of the MDM2–DAXX–HAUSP complex, thereby enhancing the p53-mediated DNA damage checkpoint [235]. Stimulated by genotoxic stress, RASSF1A interacts with MST2 and strengthens the interaction between MST2 and LATS1 to promote YAP-p73-induced apoptosis [176]. Interestingly, one study points out that by binding with SAV1, RASSF1A is capable of activating p73 independently of the Hippo pathway [182]. RASSF1A interacts with microtubule proteins and controls microtubule dynamics as well as cell polarity [236,237]. RASSF1A localizes at microtubules throughout the cell cycle, with an intense signal at the centrosomes during the interphase and early mitosis [238,239]. The overexpression of RASSF1A impairs centrosome separation, while the downregulation of RASSF1A produces centrosome overduplication [239,240].

When a cell progresses through mitosis, the RASSF1A localization pattern changes from centrosomes at early mitosis to the spindle poles in the middle of mitosis, and then RASSF1A accumulates at the spindle midzone and spindle midbody in late mitosis [238,239]. The absence of RASSF1A leads to chromosome misalignment, chromosome mis-segregation, and cytokinesis failure [48,240]. RASSF1A affects mitotic progression in multi-faceted ways. First, RASSF1A stabilizes microtubules, so cells with up-regulated RASSF1A exhibit monopolar spindles and are subject to mitotic arrest in mitosis, reminiscent of the Taxol-induced G2/M arrest phenotype [238]. Second, RASSF1A interacts with Cdc20 and interferes with the APC–Cdc20 complex-mediated degradation of Cyclin A and Cyclin B, thereby stopping the mitotic progression at the prometaphase [240]. Third, RASSF1A undergoes Aurora A/B-mediated phosphorylation and APC–Cdc20 complex-mediated ubiquitination modification, through which it affects mitotic progression [241,242,243]. RASSF1A is phosphorylated by Aurora A at T202 and S203, and the phospho-mimetic RASSF1A (T202D/S203D) disrupts its interaction with microtubules to inhibit mitotic arrest [241]. Furthermore, the phosphorylation of RASSF1A at S203 by Aurora A dissociates RASSF1A from Cdc20 to increase APC–Cdc20 activity [242]. Moreover, the Aurora A/B-induced phosphorylation of RASSF1A elicits the degradation signal, which allows for APC–Cdc20 to degrade RASSF1A [243]. Fourth, RASSF1A is also degraded by the CUL4A–DDB1 E3 complex to promote mitotic progression [244]. Fifth, RASSF1A is phosphorylated at S184 by CHK1 and phospho-mimetic RASSF1A (S184D), which results in a defective association of RASSF1A to the microtubules, abolishing the ability of RASSF1A to trigger the mitotic arrest [245]. It is worth mentioning that the Aurora B-mediated phosphorylation of RASSF1A at S203 is also essential for cytokinesis [246]. This phosphorylation is essential for RASSF1A to interact with the cytokinesis recruitment factor Syntaxin16, and phosphorylation-deficient RASSF1A (S203A) causes cytokinesis failure [246]. It is possible that RASSF1A regulates G2/M transition as well, in that RASSF1A boosts the inhibitory binding of transcription repressor p120^E4F^ to the Cyclin A2 promoter [247].

### 4.3. KIBRA in DNA Damage Checkpoint and Mitosis

In response to DNA injury, KIBRA is phosphorylated by ATM at T1006 and enhances double-strand break (DSB) repair, potentially through the non-homologous end joining (NHEJ) pathway by forming a complex with the Ku heterodimer [248]. KIBRA is phosphorylated by CDK1 at S542 and S931 in mitosis, and the CDK1-mediated phosphorylation of KIBRA is reversed by Cdc14A/B [170]. KIBRA is also phosphorylated by Aurora A/B at S539 in mitotic arrest, and the phosphorylation of S539 is counteracted by PP1 [249]. The inhibition of the CDK1- or Aurora A/B-mediated phosphorylation of KIBRA reduces the number of cells in mitosis [170,249]. In addition, KIBRA stimulates Aurora A activation, and KIBRA depletion gives rise to chromosome misalignment, chromosome mis-segregation, and multipolar spindle [250].

### 4.4. NF2 in E2F Activity, DNA Damage Checkpoint, Centrosome Dynamics, and Mitosis

NF2 overexpression decreases Cyclin D1, increases p27, represses the phosphorylation of pRb as well as E2F-dependent gene transcription, and causes G1 arrest [251,252]. More importantly, NF2 regulates cell cycle progression by interacting with a myriad of signaling molecules, including CD44, EGFR, Rac1, Ras, β-catenin, mTOR, etc., to modulate Cyclin A/D/E and p21/p27 expression [253]. Furthermore, NF2 is capable of stabilizing p53 by downregulating MDM2 at the protein level, leading to an elevated transcriptional activity of p53 and increased sensitivity to serum starvation-mediated apoptosis [254]. NF2 confers normal centrosome positioning by confining the cortical distribution of the EZM protein, Ezrin [255]. NF2 deficiency contributes to impaired Ezrin capping at the cortex, followed by centrosome de-clustering and the formation of multipolar spindles [255]. NF2 is phosphorylated by Aurora A at S518 in mitosis, which primes another phosphorylation of NF2 at T581 [256]. A non-phosphorylatable variant of NF2 (S518A/T581A) displays defective centrosome and spindle positioning at the metaphase and a longer duration from the prometaphase to the anaphase [256]. NF2 also binds and stabilizes microtubules, and the phosphorylation state of NF2 modulates its interaction with microtubules and Ezrin as well [256,257]. Moreover, NF2 may also regulate Cyclin B-CDK2 activity, in view of a finding that NF2 affects the integrity of HEI10, a cell cycle regulator that controls Cyclin B accumulation [258].

### 4.5. Ajuba in DNA Damage Checkpoint and Mitosis

Ajuba associates with the DNA binding protein RPA70 to repress ATR function during an unperturbed S phase, but it is released from RPA70 in response to DNA replication stress to allow for ATR activation [259]. Congruously, the loss of Ajuba triggers ATR-CHK1-p53 activation and results in increased cell death and cell cycle arrest [260]. Ajuba is a microtubule-associated protein that accumulates at centrosomes throughout the cell cycle and is also enriched at the spindle and spindle midzone during mitosis [261]. Ajuba is required for mitotic entry, in that it promotes Aurora A activation at centrosomes in late G2 [262]. The absence of Ajuba hinders the recruitment of γ-tubulin to the centrosomes and impinges on the spindle formation [263]. During the metaphase, Ajuba co-localizes with BUBR1 and Aurora B at kinetochores to influence the metaphase–anaphase transition [261]. In addition, Ajuba is phosphorylated at S119 and S175 by CDK1 in mitosis, and the mitotic phosphorylation of Ajuba affects the expression of many cell cycle regulators [264].

### 4.6. Zyxin in DNA Damage Checkpoint and Mitosis

Despite being a negative regulator of LATS2, Zyxin promotes UV radiation-induced apoptosis by preventing the proteasomal degradation of HIPK2, and by further increasing the HIPK2-mediated phosphorylation and activation of p53 [265]. The function of Zyxin in mitosis is still unclear, but Zyxin is an actin regulatory protein and interacts with LATS1 at the mitotic apparatus during mitosis [266]. In addition, Zyxin is phosphorylated by CDK1 in mitosis, and the mitotic phosphorylation of Zyxin is crucial for its tumorigenic activity [267].

### 4.7. NDR1/2 in E2F Activity, DNA Damage Checkpoint, and Centrosome Dynamics

Apart from regulating chromosome alignment, spindle orientation, and cytokinesis (see Section 2.2.2), NDR1/2 play parts in other phases of the cell cycle as well. Notwithstanding that there is no clear evidence suggesting that NDR1/2 regulate E2F transcriptional activity, the NDR1/2 double knockdown elevates p21 and p27 at the protein level and p21 at the mRNA level in mouse embryos [268,269]. NDR1/2 directly phosphorylate p21 at S146 and decrease p21 stability [268]. Accordingly, the NDR1/2 deficiency-induced G1 arrest is ameliorated by p21 inhibition [268]. NDR1 interacts with DNA damage sensor XPA at the nucleus, facilitates the repair of cyclobutane pyrimidine dimers, and activates the ATR-CHK1-p53 apoptotic signal in response to UV-induced DNA lesions [270]. Moreover, NDR1 binds monoufmylated H4 and assists in ATM activation through the recruitment of SUV39H1 to double-strand breaks [271]. NDR1 is also required in DNA damage-induced G2/M arrest, in part by phosphorylating Cdc25A and reducing Cdc25A stability [272]. NDR1/2 associate with centrosomes, and NDR1 is needed for centrosome amplification; targeting NDR1 to the centrosome drives centrosome overduplication in a CDK2-dependent manner [180,273].

## 5. Conclusions and Future Perspective

The cell cycle is a delicate process, and the Hippo pathway impacts it in a variety of ways. Aberrant activity of the cell cycle machinery is a common characteristic as well as a strong driving force of cancer. The dysregulated Hippo pathway acts as an accomplice to concomitantly exacerbate the uncontrolled cell cycle, potentiating the malignant and refractory cancer phenotypes. Fortunately, our cumulative endeavor in deciphering the underlying mechanisms of cell cycle abnormality in cancer have propelled CDK4/6 inhibitors and PARP inhibitors into the clinics for certain types of cancers, and many other inhibitors targeting CDKs (e.g., CDK2, CDK1, CDK7), cell cycle checkpoints (e.g., ATM/ATR, CHK1/2), microtubule-associated motor protein (e.g., kinesin-5), and mitotic kinases (e.g., Aurora A/B, PLK1), are being developed and tested in clinical trials [19,27]. However, a good cancer therapy entails efficiency and specificity, as well as prevention of resistance. The first two points concern the identification of effective, specific, and easily targeted cancer molecules; the development of specific, efficient, and potent inhibitors; the design of tumor-targeted prodrug; and the creation of effective drug delivery systems. Given that a deregulated Hippo pathway (e.g., LATS1/2 and YAP) is associated with resistance to CDK4/6 inhibitors for ER-positive and HER2^-^negative breast cancer [274], there is reason to believe that LATS1/2-mutated or YAP-amplified patients would be more susceptible to CDK4/6 inhibitor-mediated drug resistance, and therefore more likely to benefit from the combinatorial therapy of CDK4/6 and YAP inhibitors. In fact, as indicated in Section 3.3 and Section 3.5, LATS1/2 and YAP are all involved in E2F activity, in which LATS2 represses E2F activation by facilitating DREAM complex assembly, while YAP directly increases E2F-induced gene transcription. Hence, LATS2 suppression or YAP activation will attenuate the effect of CDK4/6 inhibitors and lead to drug resistance. This case exemplifies that understanding how the Hippo pathway affects cell cycle regulation could provide a basis for improving anti-cancer treatment. Considering the intense connections between cell cycle regulation and the Hippo pathway, future work is warranted to explore how targeting the cell cycle machinery, together with the Hippo pathway components, would produce synthetic lethality and prevent drug resistance in cancers. Bestowed with advanced technologies and individualized medicine, we are fueling the bench-to-bedside transition at an ever-increasing speed, increasing the likelihood of reaching the goal of more effective cures for cancer.

## Figures and Tables

**Table 1:** The conservation of the Hippo pathway in mitosis.

	*S. cerevisiae* (MEN)	*S. cerevisiae* (RAM)	*S. pombe* (SIN)	*D. melanogaster*	Mammals
The Ste20-like protein kinase	Cdc15	Kic1	Cdc7	Hpo	MST1/2
The scaffold protein	Nud1	Tao3	Cdc11-Sid4-Ppc89 complex	Sav	SAV1 or Furry
The NDR protein kinase	Dbf2/20	Cbk1	Sid2	Wts	LATS1/2 or NDR1/2
The adaptor protein	Mob1	Mob2	Mob1	Mats	MOB1A/B
The effector protein	Cdc14	Ace2	Clp1	Yki	YAP/TAZ
Function in mitosis	Mitotic exit	Cytokinesis	Mitotic exit and cytokinesis	Mitotic progression and cytokinesis	Mitotic progression and cytokinesis

**Table 2:** The mammalian Hippo pathway in the cell cycle.

	E2F Activity	G1 Tetraploidy Checkpoint	DNA Synthesis	DNA Damage Checkpoint	Centrosome Dynamics	Mitosis
MST1/2		Prevents polyploidization		Promotes DNA repair and DNA damage-induced apoptosis	Centrosome duplication and centrosome separation	Chromosome alignment and spindle formation
SAV1		Prevents polyploidization		Promotes DNA damage-induced apoptosis	Facilitates centrosome separation	Spindle formation
LATS1/2	Promotes DREAM complex assembly to repress E2F	Prevents polyploidization, enhances G1 checkpoint	Affects DNA synthesis initiation	Promotes DNA repair and DNA damage-induced apoptosis	Prevents centrosome overduplication	SAC, chromosome alignment and segregation, cytokinesis
MOB1A/B					Centrosome duplication, separation	Spindle orientation and cytokinesis
YAP/TAZ	Activates E2F and CDK2/4/6, suppresses p21/p27	Stimulates polyploidization	Promotes transcription of DNA synthesis genes	Enhances DNA repair, promotes or suppresses DNA damage-induced apoptosis		SAC, chromosome alignment and segregation, spindle orientation, and cytokinesis
TAOKs				Triggers DNA damage-induced G2/M arrest		Mitotic cell rounding, chromosome alignment and segregation, spindle orientation, cytokinesis
RASSF1A	Decreases Cyclin D-CDK4, increases p27			Promotes DNA repair and DNA damage-induced apoptosis	Inhibits centrosome separation	Chromosome alignment and segregation, cytokinesis
KIBRA				Promotes DNA repair		Chromosome alignment and segregation
NF2	Decreases Cyclin D/E, increases p21/p27			Promotes DNA damage-induced apoptosis	Centrosome position	Regulates spindle positioning
Ajuba				Represses DNA damage-induced apoptosis/arrest		Regulates spindle formation
Zyxin				Promotes DNA damage-induced apoptosis		
Furry						Chromosome alignment and spindle formation
NDR1/2					Centrosome duplication	Chromosome alignment, spindle orientation, and cytokinesis
